# The Impact of Psychopathology Associated With Childhood Trauma on Quality of Life in Portuguese Adolescents: A Two-Wave Longitudinal Study

**DOI:** 10.3389/fpsyt.2021.650700

**Published:** 2021-10-01

**Authors:** Ricardo Pinto, Maria Vieira De Castro, Laura Silva, Inês Jongenelen, Angela Maia, Alytia A. Levendosky

**Affiliations:** ^1^HEI-Lab: Digital Human-Environment Interaction Lab, Faculty of Psychology, Education and Sports, Universidade Lusófona do Porto, Porto, Portugal; ^2^Institute of Psychology and Educational Sciences, Lusíada University of Porto, Porto, Portugal; ^3^School of Psychology, University of Minho, Braga, Portugal; ^4^Department of Psychology, College of Social Science, Michigan State University, East Lansing, MI, United States

**Keywords:** adolescents, adversity, quality of life, psychopathology, trauma, social support

## Abstract

**Introduction:** The aim of this study was to explore the mediating effect of psychopathology between childhood adversity and trauma and quality of life (QOL) in adolescents. The second aim of the study was testing the moderation by social support of this mediation effect.

**Methods:** Self-reports of childhood adversity and trauma, QOL, social support, and psychopathology were collected from 150 Portuguese adolescents' who had been exposed to at least one traumatic event or one childhood adversity (*M*_age_ = 16.89, *SD* = 1.32). The surveys were administered at two time points with an approximate time interval of 1 year.

**Results:** Indirect effects were observed for depression (*B* = −0.33, CI [−0.62, −0.11]), somatization (*B* = −0.52, CI [−0.82, −0.23]), and post-traumatic stress symptoms (PTSS) (*B* = −0.23, CI [−0.45, −0.01]), but not for anxiety (*B* = 0.20, CI [−0.08, 0.50]). A moderated mediation was found between social support and depression (*B* = −0.10, CI [−16, −0.04]), and PTSS (*B* = 0.03, CI [−0.1, −0.05]), but not for somatization (*B* = −0.02, CI [−0.8, 0.05]).

**Conclusions:** We found that depression and somatization were strong mediators of the relationship between adversity/trauma and QOL, whereas PTSS was moderately mediated this relationship. Anxiety did not mediate this relationship. The moderated-mediation effect of social support was only found for depression and PTSS. The improvement of QOL in adolescents exposed to childhood adversity and trauma should include the assessment of psychopathology symptoms and social support, with the aim of identifying risk and protective factors.

## Introduction

One of the most consistent findings in mental health field is that childhood adversity and trauma are strong determinants of mental health problems throughout the lifespan (1, 2). Childhood adversity and trauma have been related to several later psychiatric disorders, including depression, anxiety disorders, and post-traumatic stress symptoms (3). Maltreated children are especially at risk for developing insecure/disorganized attachments with their primary caregiver (4), which in turn may serve as an underlying mechanism through which children with maltreatment experiences develop future psychopathology (3). In addition to psychopathology, an outcome that is frequently ignored by researchers, but very important to individuals, is quality of life (QOL). QOL is a broader construct that encompasses different aspects of life and is defined here as the perceptions of physical, emotional, and social functioning which are the core dimensions of health as delineated by the World Health Organization (5), as well as the school functioning (6, 7). This study examines whether psychopathology may be a mechanism through which childhood adversity and trauma affects quality of life for adolescents.

Children who experience adversity and trauma tend to develop negative expectations regarding the availability and trustworthiness of others, as well as mental representations of the self as incompetent and unworthy (3). From a developmental psychopathology perspective (8), adverse childhood experiences and traumas are proximal risk factors that occurred in critical stages during the childhood that interfere with a child's normal development and commence a trajectory of vulnerability. The common sequelae of childhood maltreatment include representations of the self as incompetent and unworthy, neurobiological changes that produces social information processing biases, altered patterns of emotional reactivity and emotion regulation, and reward processing (9) all serve to leave children more vulnerable to later psychopathology (10). More specifically, they may be particularly sensitive to developing psychopathology during adolescence, as it is a sensitive period for negative interpersonal events due to the intensified emotional potency of interpersonal interactions during this developmental period (11, 12). Psychopathology during adolescence has consequences for physical, cognitive and emotional functioning (12–15), and academic functioning (16) as adolescents struggled to navigate their increasingly complex social worlds. These negative consequences for functioning inherently result in a worse quality of life for these adolescents. In contrast, abused children who do not develop psychopathology during adolescence are resilient and thus are likely to have a better quality of life. In other words, the relationship between childhood adversity/maltreatment and quality of life in adolescence is mediated by adolescent psychopathology.

However, to our knowledge, there is no current evidence about this mediating hypothesis. A previous systematic review of the literature between the years 1976–2006 (17) only found four studies that evaluated QOL for adult survivors of child maltreatment. Similarly, a recent systematic review found a few studies on QOL for maltreated children and adolescents (18). Specifically, Gospodarevskaya (19), in a sample with 993 adolescents who experienced childhood sexual abuse, found that psychopathology, namely post-traumatic stress, corresponded to significantly lower levels of quality of life. Additionally, Chan (20), in a sample of 18,341 adolescents, found that child victims of violence were more likely to report PTSD and depressive symptoms, self-harm ideation, and poor QOL. However, neither of these studies examined the potential mediation effect of psychopathology between the relationship of childhood adversity and trauma and subsequent QOL in adolescents.

One factor that can be protective against maltreatment and trauma is social support (21). According to the developmental psychopathology perspective (8), social support is one of the most important protective factors within the broader constellation of risks and vulnerabilities that children experience. Social support can be stress-buffering as a protective factor when children encounter later developmental challenges. Among children exposed to adversity, social support can buffer the stress caused by marital conflict, domestic violence, and parental affective disturbances (22), and prevent the development and maintenance of psychopathology later in adolescence. While social support may aid children in coping and help them to develop better social skills, the absence of it may lead to isolation from family and peer groups or dysfunctional social relationships, that can contribute to the development of psychopathology, with negative impact on QOL (23).

Therefore, the aim of this study was to explore the mediating effect of psychopathology between childhood adversity and trauma and QOL in adolescents. The second aim of the study was testing the moderation by social support of this mediation effect. Data for this study came from surveys administered in two separate waves of data collection, with an approximate time interval of 1 year, to examine the distal effects of childhood adversity and trauma (time 1, retrospectively assessed), the mediation effect of psychopathology symptoms (time 1), on outcome variable QOL (time 2).

## Methods

### Participants

We contacted 232 adolescents living in institutions run by Child Protective Services (CPS) and 271 adolescents attending vocational schools. We chose these two places for recruitment because of the high-risk nature of the adolescent population involved in these programs. Our inclusion criteria included adolescents between the ages of 13 and 17. This 13-year age criterion is due to the minimum age to enter a vocational school before which the student must have completed 9 years of schooling. Another inclusion criterion was that the adolescent had to report being exposed to at least one traumatic event or one childhood adversity. Exclusion criteria was having intellectual disability that could compromise the understanding of the informed consent and the protocol questions. We obtained written informed consent from both adolescents and their parents or legal guardians to participate in the study from 210 adolescents, but only 183 met the criteria to participate in this study, mean of the age of 16 years old (*M* = 15.99, *SD* = 1.25), ranged between 13 and 18 years old.

At the second wave, ~1 year after the first assessment, the expected time to demonstrate effects on the quality of life (24–26), we attempted to recontact all the study participants and were successfully in finding 150 of them. The answer to whether this was enough time for any difference in the quality of life to be evident must be clearly stated and supported. Thus, the final sample of the current study is 150 participants. The mean of the age of this sample was 17 years old (*M* = 16.89, *SD* = 1.32), ranging between 14 and 19 years old, including 67 (44.7%) males and 83 (55.3%) females. In total, 73 (48.7%) of the adolescents had been previously identified by CPS due to exposure to child abuse, neglect, or other family dysfunction. In terms of mothers' and fathers' educational levels, 47 (31.3%) fathers had elementary school, 48 (32%) middle school, 45 (30%) high school, and 8 (5.3%) university degree; 42 (28%) mothers had elementary school, 70 (46.7%) middle school, 32 (21.3%) high school, and 6 (4%) university degree. In relation to the family income, most of the adolescents (*n* = 80, 53.4%) reported a monthly household income between $270 and $800 U.S. dollar, corresponding to low socioeconomic status. There were no differences between those who participated in the second wave from those who did not participate in terms of age [*t*_(187)_ = 1.57, *p* = 118], trauma exposure [*t*_(186)_ = 1.74, *p* = 08], and PTSD symptoms [*t*_(187)_ = 0.407, *p* = 684]. However, we found differences in terms of childhood adversity and trauma exposure, [*t*_(186)_ = 3.07, *p* = 002]. The ones who dropped out had more childhood adversity and traumas (*M* = 9.32; SD = 3.53) compared to those who participated in both assessments (*M* = 7.02; *SD* = 4.25).

### Procedure

The present study is part of a larger longitudinal research project on the impact of traumatic events on adolescents in the North of Portugal (27–29). All procedures performed in this study were in accordance with the APA ethical standards. The study was approved by the ethics committee of the [removed for blind review]. In order to recruit participants, 14 institutions run by Child Protective Services (CPS) and 16 vocational schools were contacted. The CPS institutions and vocational schools were initially contacted by e-mail and then by telephone with the purpose of scheduling an initial interview. Upon authorization by these institutions, the data collection started. The adolescents who agreed to participate were given more detailed information about the study and delivered a written informed consent to be signed by parents or legal guardians, in order to allow participation in the study. The informed consent was obtained before beginning administration of the questionnaires. The questionnaires were administered by three trained psychologists in a private room for the purposes of confidentiality. In addition, adolescents were able to send an email to the researchers if they wished to have access to their results or felt the need to talk with the researchers about any information contained in the protocol.

### Measures

#### A Demographic Questionnaire

A demographic questionnaire, composed of multiple-choice questions, was used to collect information about age, gender, and family information (i.e., number of household, educational level of the parents, income, and housing changes). This measure also included items about current residence (e.g., with parents, with grandparents, institution, etc.) and if the adolescent ever was identified by Child Protective Services (CPS).

#### The Life Events Checklist for DSM 5

The Life Events Checklist for DSM 5 (LEC-5) [(30); Portuguese version: (removed for blind review)] is a self-report measure developed to evaluate traumatic events in an individual's life according to the DSM 5. It evaluates exposure to 16 potentially traumatic events (e.g., natural disasters, accidents, and sexual assault) and includes an additional item where respondents could report another traumatic event that was not listed in the 16 previous items. For each event, respondents indicate their level of exposure (e.g., direct experience; witnessing). Additionally, participants were asked to select the most traumatic event they had experienced, and to indicate how long ago it had happened. The LEC-5 has been demonstrated to be a good measure of exposure to traumatic events and has convergent validity with measures of trauma related psychopathology (31). The study of the original version showed Pearson coefficients ranging from 0.44 to 0.48 between LEC and PTSD symptom severity, and 0.27 and 0.32 between LEC and measures of anxiety and depression, respectively. The Pearson coefficients for the present sample are reported in Table 1.

**Table 1:** Means and standard deviation of key measures.

**Variables**	**Total sample (*****N*** **=** **150)**			
	***M***	***SD***	**Minimum**	**Maximum**
Quality of life	20.40	13.04	0	59
Cumulative adversity	7.49	4.20	0	19
PTSD symptoms	22.89	18.20	0	67
Depression symptoms	6.31	5.31	0	24
Anxiety symptoms	4.13	3.92	0	18
Somatic symptoms	4.15	4.42	0	20
Social support	43.23	8.71	20	78

*It was used the total score for all variables*.

#### Adverse Childhood Experiences Study Questionnaire

Adverse Childhood Experiences Study Questionnaire [ACE: (32); Portuguese version: (removed for blind review)] is a retrospective self-report measure which assesses the occurrence of adverse experiences in childhood. This questionnaire includes detailed information on 10 adverse childhood experiences (e.g., emotional abuse, physical abuse, sexual abuse, exposure to domestic violence, substance abuse in the family environment, divorce or parental separation, family member, mental illness or suicide, physical neglect and emotional neglect), organized into two areas: children's experiences and household dysfunction [removed for blind review]. Responses range from 0 (never) to 5 (very often), with the exception of sexual abuse, for which a dichotomous response (yes or no) was given and all items were dichotomized based on how often the experiences occurred [see (32)]. We then computed a total score of the adverse experiences exposure for each subject ranged from zero to 10. The study of the original scale demonstrated good test-retest reliability (33). In the present study, the internal consistency was 0.82.

#### The Child PTSD Symptom Scale—V

The Child PTSD Symptom Scale—V (CPSS-V) [(34); Portuguese version: (removed for blind review)] is a self-report measure that aims to assess the severity of PTSD symptoms presented in the past month by children and adolescents after having being exposed to a traumatic event. The inventory comprises 20 items corresponding to PTSD symptoms according to the criteria of the DSM-5. Participants rate the frequency that they experience each symptom using a 4-point Likert scale, ranging from 0 (never), to 4 (6 or more times per week/ almost always), yielding a total score of 80 possible points, indicating PTSD symptom severity. Examples of items are “Having feelings in your body when you remember what happened (for example, sweating, heart beating fast, stomach or head hurting)” and “Trying not to think about, talk about, or have feelings about it [the event].” The original study demonstrated an excellent internal consistency for the total score (α = 0.92), and the internal consistency for each scale ranged from acceptable to good, meaning: for criterion B (α = 0.81), for criterion C (α = 0.63); for criterion D (α = 0.86) and for criterion E (α = 0.72) (35).

#### Brief Symptom Inventory

Brief Symptom Inventory (BSI) [(36); Portuguese version: (37)] is a well-established self-report instrument to assess psychological distress. The BSI comprises 53 items on a 5-point rating scale that ranges from 0 (not at all) to 4 (extremely). The inventory includes nine symptom dimensions: somatization, obsessive compulsivity, interpersonal sensitivity, depression, anxiety, hostility, phobic anxiety, paranoid ideation, and psychoticism. For the purposes of the present study, we only used the depression, anxiety and somatization subscales. Example of items are “Feeling no interest in things” and “Feeling tense or keyed up.” Higher scores reflect higher depression, anxiety, and somatization symptoms. The studies of the original scale have demonstrated good internal consistency for each of the scales, ranging from 0.71 to 0.85 (36). The internal consistency of the present sample was 0.87 for depression, 0.86 for anxiety, and 0.83 for somatization.

#### The Scale of Satisfaction With Social Support for Children and Adolescents

The Scale of Satisfaction with Social Support for Children and Adolescents (ESSS-CA) (38) is a self-report measure that assesses the perception of social support in children and adolescents. The original version includes 15 items, but we used a reduced version of 12 items to assess two dimensions of social support, as satisfaction and activities, using a five-point Likert scale ranging from “totally agree” to “strongly disagree.” Example of items are “I'm satisfied with the number of friends I have” and “When I need to vent, I can easily find someone to do it.” A total score was computed by summing all items, with higher scores indicating a greater degree of social support satisfaction. The study of the original scale demonstrated acceptable to good internal consistency, between 0.69 and 0.84 (38). The internal consistency for the present sample was 0.75.

#### Pediatric Quality of Life Inventory Version 4.0

Pediatric Quality of Life Inventory Version 4.0 [(7); Portuguese version: Ferreira et al. (39)]. The 23-item PedsQL™ 4.0 Generic Core Scales encompass: (1) Physical Functioning (8 items), (2) Emotional Functioning (5 items), (3) Social Functioning (5 items), and (4) School Functioning (5 items). The self-report version for 13–18 years was used in this study. The instructions ask how much of a problem each item has been during the past 1 month. A 5-point Likert response scale is utilized across child self-report (0 = never a problem; 1 =almost never a problem; 2 = sometimes a problem; 3 =often a problem; 4 = almost always a problem). Items are reverse-scored and linearly transformed to a 0–100 scale (0 = 100, 1 = 75, 2 = 50, 3 = 25, and 4 = 0), so that higher scores indicate better QOL. Scale scores were computed as the sum of the items divided by the number of items answered (thus accounting for missing data).

### Data Analysis

Data analyses were carried out using the SPSS version 20 for Windows. We calculated a composite index (*Z*-scored and then added) of childhood adversity and trauma as the independent variable. Mediation analyses were conducted to determine direct and indirect effects with 5,000 bootstrapped samples and bias-corrected 95% confidence intervals (BCa CI) using the SPSS PROCESS macro (Model 4) (40). The moderated mediation model was analyzed by Hayes's PROCESS macro (Model 7). No correction was made for missing values in the reported analyses considering the low incidence observed.

## Results

Descriptive data of key measures are presented in Table 1. Quality of life was significantly associated with all study variables (Table 2).

**Table 2:** Correlations of key measures.

**Variables**	**1**	**2**	**3**	**4**	**5**	**6**	**7**	**8**	**9**
1. Quality of life	–								
2. Cumulative adversity	−0.44^***^	–							
3. PTSD criterion B	−0.44^***^	0.31^***^	–						
4. PTSD criterion C	−0.29^**^	0.24^**^	0.62^***^	–					
5. PTSD criterion D	−0.39^***^	0.34^***^	0.75^***^	0.58^***^	–				
6. PTSD criterion E	−0.42^***^	0.35^***^	0.70^***^	0.53^***^	0.73^***^	–			
7. Depression symptoms	−0.48^***^	0.43^***^	0.57^***^	0.38^***^	0.62^***^	0.58^***^	–		
8. Anxiety symptoms	−0.41^***^	0.36^***^	0.52^***^	0.36^***^	0.57^***^	0.59^***^	0.66^***^	–	
9. Somatic symptoms	−0.47^***^	0.44^***^	0.51^***^	0.38^***^	0.59^***^	0.60^***^	0.56^***^	0.77^***^	–
10. Social Support	0.39^***^	−0.45^***^	−0.17	−0.16	−0.24^**^	−0.25^**^	−0.35^***^	−0.19^*^	−0.20^*^

* *p < 0.05, two-tailed*.

** *p < 0.01, two-tailed*.

*** *p < 0.001*.

### Testing the First Hypothesis—Mediation Model

The cumulative adversity was input into the model as predictor, the PTSD symptoms, somatization, depression, and anxiety symptoms were included as mediators, and QOL as outcome variable. The overall regression model testing the effect of cumulative adversity on quality of life, with proposed mental health mediators was statistically significant, *F*_(7,159)_ = 22.09, *p* < 0.001, accounting for 49% of the variance. Age and gender were included as covariates in the model.

### Path a: Effect of Cumulative Adversity on Mediators

The findings indicated a statistically significant effect of the cumulative adversity on depression (*B* = 0.47, SE = 0.10, *p* < 0.001, CI [0.28, 0.66], anxiety (*B* = 0.37, SE = 0.07, *p* < 0.001, CI [0.22, 0.52], somatization (*B* = 0.45, SE = 0.08, *p* < 0.001, CI [0.29, 0.62], and PTSS (*B* = 0.08, SE = 0.02, *p* < 0.001, CI [0.05, 0.12]).

### Path b: Effect of Mediators on Quality of Life

Several of the mediators, depression (*B* = −0.83, SE = 0.30, *p* < 0.01, CI [−1.43, −0.23], somatization (*B* = −0.89, SE = 0.40, *p* < 0.05, CI [−1.68, −0.23], and PTSS (*B* = −0.23, SE = 0.09, *p* < 0.05, CI [−0.45, −0.02]), significantly predicted quality of life. However, anxiety was not a significant mediator (*B* = 0.47, SE = 0.48, *p* = 0.33, CI [−0.49, 1.42].

### Paths c and c′: Direct and Indirect Effects on Quality of Life

**Figure 1:**
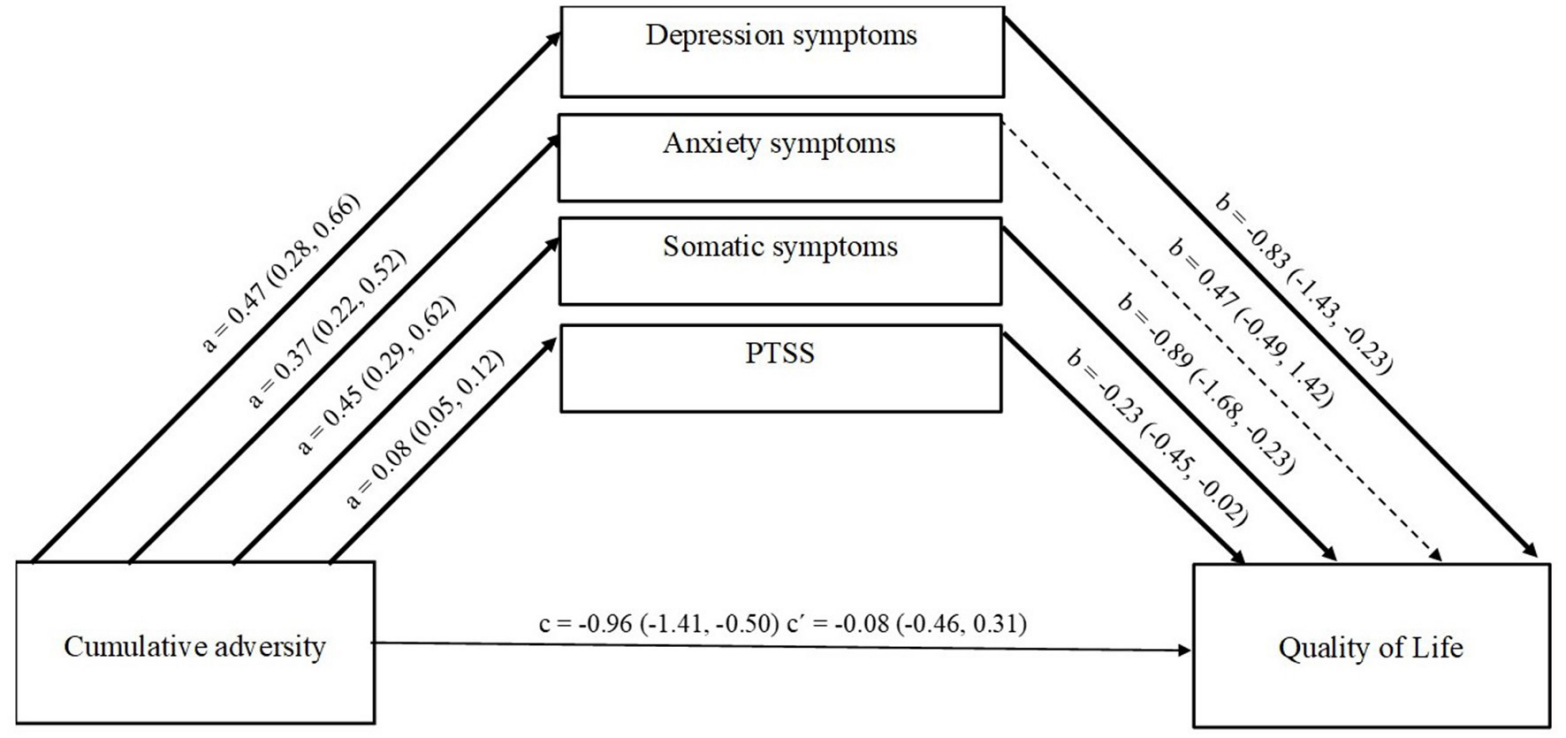
Effect of multiple mediators on the relationship between Cumulative Adversity and Quality of Life. a, effect of TLEs on mediators; b, effect of mediators on Quality of Life; c, direct effect of TLEs on Quality of Life; c′, indirect effect of TLEs on Quality of Life through the four mediators. Values are unstandardized coefficients and Bootstrap Confidence Intervals.

The total effect of the cumulative adversity on quality of life was significant (*B* = −0.96, SE = 0.23, *p* < 0.001, CI [−1.41, −0.50]), but the effect was completely lost (*B* = −0.08, SE = 0.20, *p* = 0.69, CI [−0.46, 0.31]), when indirect effects were included for depression (*B* = −0.33, BSE = 0.13, CI [−0.62, −0.11], somatization (*B* = −0.52, BSE = 0.15, *p* < 0.001, CI [−0.82, −0.23], and PTSS (*B* = −0.23, BSE = 0.11, CI [−0.45, −0.01]). However, this was not true for the indirect effects of anxiety symptoms, (*B* = 0.20, BSE = 0.14, CI [−0.08, 0.50]) (see Figure 1).

### Testing for the Moderated Mediation

**Figure 2:**
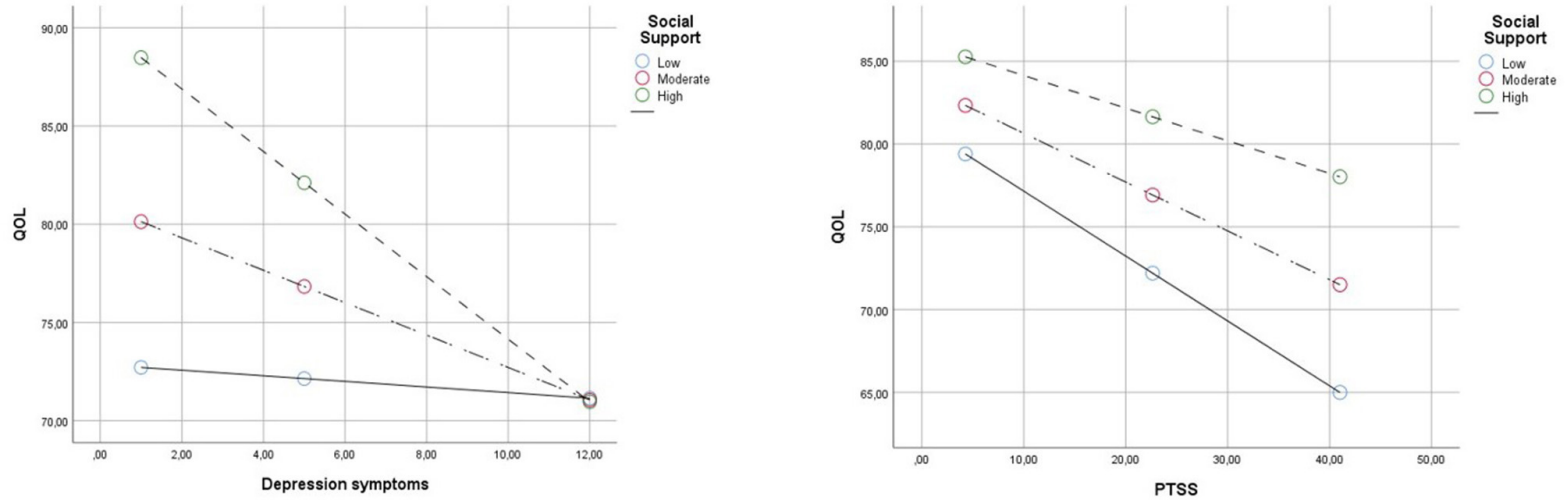
Simple slopes representing the relationship between PTSS/Depression Symptoms and QOL scores at −1 SD, mean, and +1 SD values of social support scores.

In Hypothesis 2, we expected that social support would buffer the indirect effects between psychopathology and quality of life. Social support (*B* = 0.46, SE = 0.12, *p* < 0.001, CI [0.20, 0.71]) was a significant predictor of quality of life. An indirect effect (moderating effect) of the highest order interaction between the depression and social support was significant (*B* = −0.10, SE = 0.03, *p* < 0.001, CI [−16, −0.04]), but only at moderate, CI = (−1.43, −0.26), and high, CI = (−2.63, −0.79), levels of social support (see Figure 2). An indirect effect between PTSS and social support was significant (*B* = −0.03, SE = 0.01, *p* = 0.01, CI [−0.1, −0.05]), at low, CI = (−0.54, −0.25), moderate, CI = (−0.42, −0.17), and high, CI = (−0.37, −0.02), levels of social support. No indirect effect was found for somatization and social support (*B* = −0.02, SE = 0.03, *p* = 0.66, CI [−0.8, 0.05]).

## Discussion

This study explored the mediation effect of psychopathology between childhood adversity and trauma and quality of life in adolescents. The second aim was testing the moderation of the mediation effect through the social support. Data for this study come from surveys administered in two separate waves of data collection, with an approximate time interval of 1 year.

The first hypothesis of a mediation effect of psychopathology symptoms between childhood adversity and trauma and QOL was confirmed, except for anxiety symptoms. Depression and somatization had a large effect and PTSS a medium effect as mediators. Following this hypothesis, a previous study found that psychopathology was a mediator between obesity and QOL among children and adolescents (23), but the authors did not explore the types of psychopathology. According to the authors, it is reasonable to assume that the negative emotions and maladaptive schemas caused by having psychological problems will affect the adolescent's QOL. The current findings are important in suggesting that impaired QOL in adolescents exposed to adverse childhood experiences and trauma is related to their psychopathology symptoms and not their violence exposure *per se*. Considering a developmental perspective, aspects of environmental context that are difficult to modify, such as childhood adversity, can work as distal risk factors for the emergence of psychopathology (41), which in turn, psychopathology may have the role of proximal factor for the impaired QOL. Therefore, the advantage in identifying these proximal risk factors for the impaired QOL is because they are often easier to modify than distal risk factors (42). Additionally, this finding could mean that adolescents who were maltreated and did not develop psychopathology are resilient and thus the childhood adversity and trauma had no impact on quality of life. According to developmental models, individual differences in pathways toward normal and abnormal functioning arise from the individual's particular profile of risk and protective experiences as accumulated through development (43, 44). In practice, increasing the quality of life in adolescents involves reducing psychopathology, since it is not possible to prevent them from being previously exposed to trauma and violence.

This perspective emphasizes that childhood adversity and trauma are not directly causally linked with QOL during the adolescence, but are instead mediated by the risk and moderated by protective factors that follow. Based on the second hypothesis, social support moderated the mediation effect of depression and PTSS, but not for somatization. When examining the moderation of the mediation effect of depression, it was noticeable in the simple slope that at high levels of depression, social support did not attenuate the impact on QOL, while it did for high levels of PTSD. These puzzling findings emphasize the importance of further studying the role of multiple types of psychopathology as mechanisms underlying the associations between childhood adversity and trauma and QOL, identifying the different effects that can have. These different findings between depression and PTSD may be because the social support is not static, but is given and received in the context of relationships. It depends on the individual's ability to create and maintain supportive social relationships, to interact with the others, and to ask for help when needed, and importantly, high levels of depression would likely cause an adolescent to alienate others and/or resist social support and to become further isolated within their families, peer groups, and communities, affecting their own QOL.

The ability to receive social support is founded on the early parent-child relationships by creating provisional representations about the quality of support they can expect from others they encounter (22). However, within abusive families, it may shatter the basic assumptions about themselves and the others, resulting in the development of self-other schemas of fear, insecurity, unpredictably, distrust, self-blame, and emotions of guilt, shame, and anger. These negative basic beliefs and emotions may compromise the adolescent's ability to seek help, to enjoy support and caring that demands trust in others, which in turn will predispose adolescents for development of psychopathology and subsequently worse QOL. This explains why the adolescents with lower levels of social support have higher levels of PTSS and lower levels of QOL. On the other hand, social support is not limited to parents. Especially in adolescence, there is an extension of social support within natural social networks, such as peer relationships, teachers, extended family, neighbors, coaches, and mentors. Adolescents who have been able to benefit from their extant support are those for whom social support may be protective and thus helps to prevent the development of psychopathology, leading to a better quality of life, despite having been victims of childhood adversity and trauma.

A few limitations of this study should be noted. First, the study relied on a non-representative sample of adolescents. Future studies should try to include representative samples of adolescents, for example by obtaining funding for national projects and having partnerships with entities that support young people at risk. Second, our longitudinal study is limited by only having two waves of data collection, 1 year apart. In future research, it is necessary to replicate our findings over a more extended longitudinal study, thus allowing us to examine potential non-linear effects. Third, the variables in this study were measured in terms of self-report questionnaires. Additional research should consider the use of structured clinical interviews and multiple sources (e.g., parents, teachers, and observations).

Despite these limitations, the present study makes a valuable contribution to the literature on QOL in adolescents with history of childhood adversity and trauma. Prevention programs should include the assessment of adversity and trauma in their protocols, together with the assessment of psychopathology symptoms and social support, with the aim of identifying risk and protective factors and thus preventing the decrease in QOL in adolescents. Nevertheless, research should be conducted to elucidate the puzzling finding exploring why some types of psychopathology may function as different mechanisms in the relationship between adversity and trauma and subsequent quality of life in adolescents.

## Data Availability Statement

The raw data supporting the conclusions of this article will be made available by the authors, without undue reservation.

## Ethics Statement

The studies involving human participants were reviewed and approved by Ethics Committee of the University Lusófona of Porto. Written informed consent to participate in this study was provided by the participants' legal guardian/next of kin.

## Author Contributions

LS collected the data. RP, MD, IJ, and AM analyzed and interpreted the data. RP and MD wrote the current version of the manuscript with critical revision of AL. All authors contributed substantially to the conception and design of the study, critical revision, and approved the final version of the article.

## Conflict of Interest

The authors declare that the research was conducted in the absence of any commercial or financial relationships that could be construed as a potential conflict of interest.

## Publisher's Note

All claims expressed in this article are solely those of the authors and do not necessarily represent those of their affiliated organizations, or those of the publisher, the editors and the reviewers. Any product that may be evaluated in this article, or claim that may be made by its manufacturer, is not guaranteed or endorsed by the publisher.
